# Selective suppression of bacterial contaminants by process conditions during lignocellulose based yeast fermentations

**DOI:** 10.1186/1754-6834-4-59

**Published:** 2011-12-20

**Authors:** Eva Albers, Emma Johansson, Carl Johan Franzén, Christer Larsson

**Affiliations:** 1 Department Chemical and Biological Engineering, Chalmers University of Technology, 412 96 Gothenburg, Sweden; 2 Processum Biorefinery Initiative, 891 80 Örnsköldsvik, Sweden

**Keywords:** ethanol, fermentation, contaminants, bacteria, lignocellulosic, yeast, *Saccharomyces*

## Abstract

**Background:**

Contamination of bacteria in large-scale yeast fermentations is a serious problem and a threat to the development of successful biofuel production plants. Huge research efforts have been spent in order to solve this problem, but additional ways must still be found to keep bacterial contaminants from thriving in these environments. The aim of this project was to develop process conditions that would inhibit bacterial growth while giving yeast a competitive advantage.

**Results:**

Lactic acid bacteria are usually considered to be the most common contaminants in industrial yeast fermentations. Our observations support this view but also suggest that acetic acid bacteria, although not so numerous, could be a much more problematic obstacle to overcome. Acetic acid bacteria showed a capacity to drastically reduce the viability of yeast. In addition, they consumed the previously formed ethanol. Lactic acid bacteria did not show this detrimental effect on yeast viability. It was possible to combat both types of bacteria by a combined addition of NaCl and ethanol to the wood hydrolysate medium used. As a result of NaCl + ethanol additions the amount of viable bacteria decreased and yeast viability was enhanced concomitantly with an increase in ethanol concentration. The successful result obtained via addition of NaCl and ethanol was also confirmed in a real industrial ethanol production plant with its natural inherent yeast/bacterial community.

**Conclusions:**

It is possible to reduce the number of bacteria and offer a selective advantage to yeast by a combined addition of NaCl and ethanol when cultivated in lignocellulosic medium such as wood hydrolysate. However, for optimal results, the concentrations of NaCl + ethanol must be adjusted to suit the challenges offered by each hydrolysate.

## Background

Contamination by bacteria in industrial scale yeast fermentations is a huge problem with serious economic consequences. Such operations are not carried out under aseptic conditions and *Lactobacilli*, which are usually considered to be the most frequent contaminants, thrive under the very same conditions as the yeast *Saccharomyces cerevisiae *[1-3]. In some conditions and for certain products the bacteria can provide added value in the form of flavor, taste, and so on, but the levels must be maintained within certain limits [4]. In other processes, such as production of biofuels like ethanol, bacterial contamination causes reductions in yield and/or productivity with a deteriorating economy of the process as a consequence. Despite massive amounts of time and effort spent on these matters, bacterial contamination is still a serious problem and a threat to the successful development of commercial bio-based fuel production. Traditional methods for keeping bacterial contaminants at a tolerable level include introduction of very low pH, for example, between 2 and 3 [5], and more modern techniques rely on the ancient knowledge that hops can provide not only a favorable taste of various beverages but also protection against bacterial decomposition of the product [6,7]. However, these methods do not always function as expected, and there is still a need to find additional ways of preventing bacteria from flourishing in these environments. Another option sometimes considered is the use of antibiotics. However, this is questionable from an economic point of view but even more important is the increasing awareness and fear of the ever-increasing spread of bacterial resistance due to massive misuse of these compounds.

This investigation was undertaken in order to develop process conditions that would present a selective advantage to the yeast while suppressing growth and product formation of bacteria. In addition, several of the conditions were selected to be relevant for so-called high gravity or high solids fermentation as this would offer high product concentration and an improved economy of the ethanol production process [8]. In order to make relevant comparisons of the selective effect on yeast and bacteria between different conditions, we isolated numerous bacterial isolates from the industrial ethanol production plant in Örnsköldsvik, Sweden from which the yeast strain used was originally isolated. The process conditions selected were enhanced levels of sugar, sodium chloride and ethanol as well as low pH. Initially these conditions were tested one by one using pure cultures of yeast and bacteria. Later on, cocultures of yeast and bacteria were studied in competition experiments and combinations of stress factors were also included. The results showed that a combination of NaCl + ethanol additions to the wood hydrolysate could suppress growth of bacteria while yeast viability and ethanol production was favored.

## Results and Discussion

In order to identify what bacterial species that should be included in the study sampling of the microbial community at an industrial ethanol production plant, Domsjö Fabriker AB in Örnsköldsvik, Sweden, were performed. The most abundant species of *Lactobacillus *seemed to be *Lactobacillus buchneri and Lactobacillus plantarum*, that is, most of the isolates obtained belonged to these two species. A full report concerning identified bacterial species and their respective growth behavior, stress tolerance and so on, will be reported in a separate publication. The species of *Lactobacillus *and *Acetobacter *that are included in this investigation were all obtained from this plant with the exception of *Lactobacillus fermentum *that was obtained from the American Type Culture Collection (ATCC; http://www.lgcstandards-atcc.org/). This latter strain was used as a reference as it had previously been investigated in a similar type of study [9].

### Viability of bacterial contaminants with or without cocultivation with yeast

**Figure 1:**
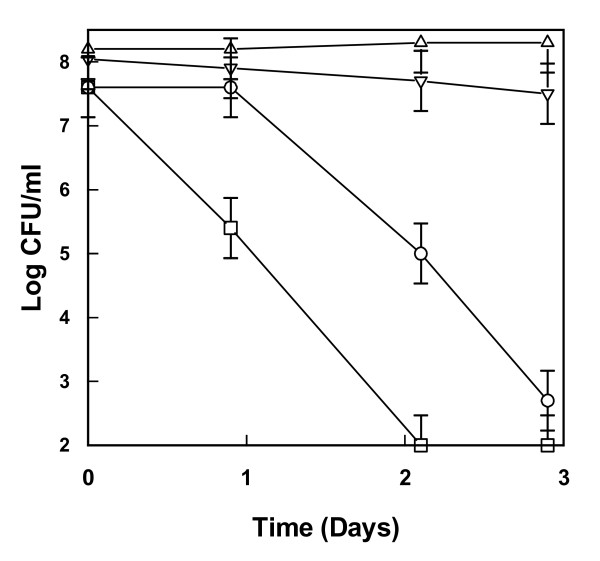
**Viability of bacteria in lignocellulosic medium**. Viability of *Lactobacillus buchneri *(circles), *Lactobacillus fermentum *(squares), *Acetobacter syzygii *(upward triangles) and *Acetobacter tropicalis *(downward triangles) inoculated into a lignocellulosic medium from chips of spruce hydrolyzed with dilute acid. The composition of the medium is described in [14]. Values represent duplicate cultures performed in falcon tubes and the error bars indicate mean and standard deviation.

Even though bacteria such as *Lactobacilli *frequently contaminate industrial yeast fermentations, none of the tested *Lactobacilli *strains included in this study could grow or even maintain its viability in the hydrolysate without the presence of yeast (Figure 1). In fact, it is worth mentioning that none of the more than 15 *Lactobacilli *isolates obtained managed to multiply in the absence of yeast (data not shown). *Acetobacter*, showed an entirely different behavior and the viability was well preserved, or even slightly enhanced, during the incubation time of 3 days (Figure 1).

**Figure 2:**
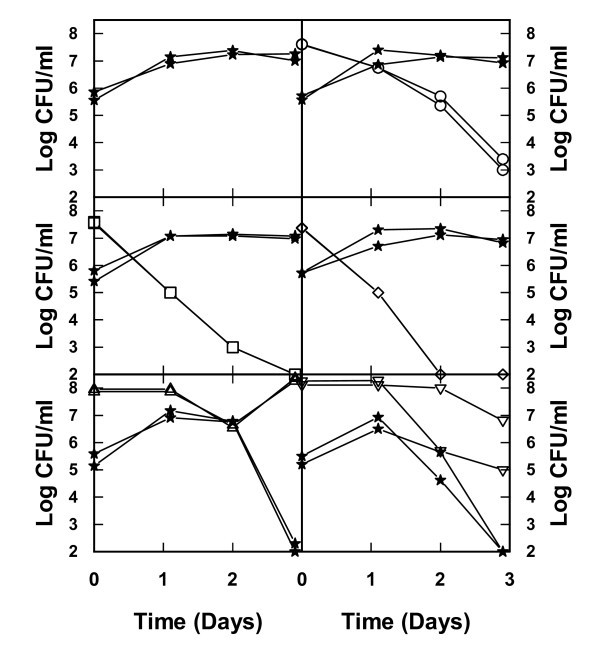
**Viability of bacteria when cocultured with yeast in lignocellulosic medium**. Viability was determined during batch cultures of the yeast *Saccharomyces cerevisiae *(filled stars) alone and in coculture with *Lactobacillus buchneri *(open circles), *Lactobacillus fermentum *(open squares), *Lactobacillus plantarum *(diamonds) *Acetobacter syzygii *(upwards triangles), and *Acetobacter tropicalis *(downwards triangles) on a lignocellulosic medium from chips of spruce hydrolyzed with dilute acid. The composition of the medium is described in [14]. In each case, results from two independent cultures performed in falcon tubes are shown.

In cocultures of bacteria and yeast there was a tendency to a slight improvement in preservation of viability of *L. fermentum *although on average the increase is less than 1 log unit (Figures 1 and 2). However, the most striking effect of these coculture experiments was the severe effect on the viability of the yeast *S. cerevisiae *from the presence of *Acetobacter *(Figure 2). Without bacteria, *S. cerevisiae *managed to grow and multiply close to five generations in the hydrolysate used, but in the presence of *Acetobacter *there was a drastic reduction in the viability of the yeast after a growth period of 1 to 2 days (Figure 2).

### The effect of nutrient supplementation (yeast extract) on viability of bacteria

**Figure 3:**
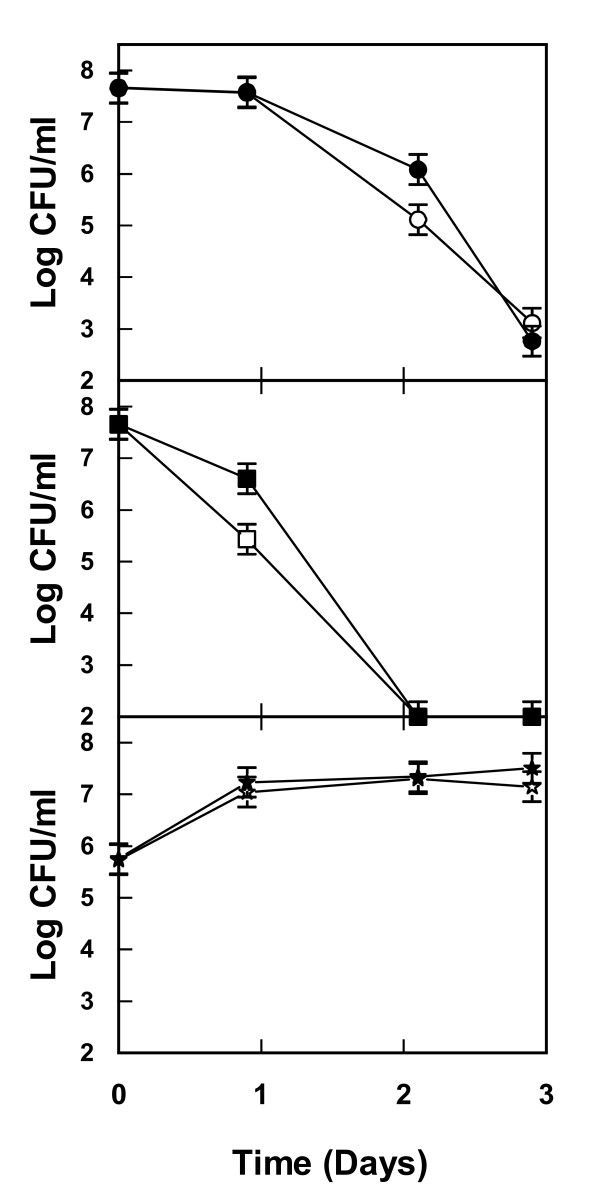
**Viability of bacteria and yeast in lignocellulosic medium with and without addition of yeast extract**. Viability of *Lactobacillus buchneri *(circles), *Lactobacillus fermentum *(squares), and *Saccharomyces cerevisiae *(stars) inoculated into lignocellulosic medium with (filled symbols) or without (open symbols) the addition of 1 g/l of yeast extract. The composition of the medium is described in [14]. Values represent duplicate cultures performed in falcon tubes and error bars indicate mean and standard deviation.

One reason for the somewhat increasing viability of *Lactobacilli *in the presence of yeast cells could be that yeast will offer some additions of essential nutrients to the bacteria [10]. To test this hypothesis we added yeast extract to the hydrolysate, but no effect whatsoever on viability of *Lactobacilli *could be detected (Figure 3). Most probably, the effect of yeast additions on viability of bacteria originate from the fact that the activity of *S. cerevisiae *will detoxify the hydrolysate [11,12] perhaps with some added value from nutrient availability.

Similarly, no effect on yeast viability and multiplication could be detected as a result of yeast extract additions to the hydrolysate (Figure 3).

### Growth and product formation during cocultivation of yeast and *Lactobacillus *or *Acetobacter*

Coculture experiments revealed large effects on growth and viability of yeast and bacteria when coexisting in the lignocellulosic medium. How did this affect metabolism and major catabolic products such as ethanol, acetate and lactic acid?

**Figure 4:**
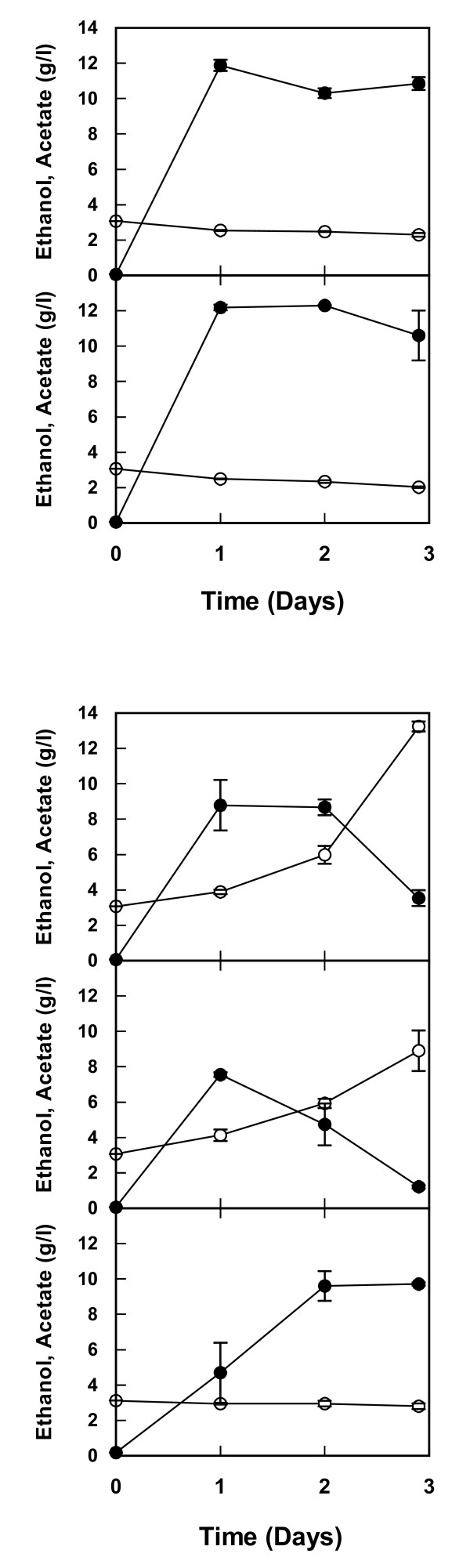
**(a) Ethanol (filled circles) and acetate (open circles) concentrations in cocultures of yeast and lactic acid bacteria**. Samples were taken from cocultures of *Saccharomyces cerevisiae *and *Lactobacillus buchneri *(upper panel) or *S. cerevisiae *and *Lactobacillus fermentum *(lower panel) inoculated in a lignocellulosic medium from chips of spruce hydrolyzed with dilute acid. The composition of the medium is described in [14]. Values represent duplicate cultures performed in falcon tubes and error bars indicate minimum/maximum values. **(b) **Ethanol (filled circles) and acetate (open circles) concentrations in cocultures of yeast and acetic acid bacteria. Samples were taken from cocultures of *S. cerevisiae *and *Acetobacter syzygii *(upper panel), *S. cerevisiae *and *Acetobacter tropicalis *(middle panel) or pure culture of *S. cerevisiae *(lower panel) inoculated in a lignocellulosic medium from chips of spruce hydrolyzed with dilute acid. The composition of the medium is described in [14]. Values represent duplicate cultures performed in falcon tubes and error bars indicate minimum/maximum values.

The ethanol concentrations reached about 12 g/l and there was no negative effect due to the presence of *Lactobacilli *(Figure 4A, 4B). Lactic acid concentrations were very low, below 0.1 g/l, throughout the duration of the experiment and no production or consumption was detected. Acetate was found at initial concentrations of about 3.0 g/l and a slow consumption resulted in final concentrations between 2.0 to 2.3 g/l (Figure 4A).

A completely different picture emerged when *S. cerevisiae *was mixed with *Acetobacter *in cocultures. In this case there was production of ethanol during the initial 24 h but this was followed by declining ethanol concentrations concomitant with an increase in acetate concentrations (Figure 4B), similar to what is normally observed during growth of *Acetobacter *[13]. Obviously, the *Acetobacter *not only has a very negative effect on the viability of the yeast (Figure 2) but also consumes a substantial part of the previously formed ethanol.

### The selective effect on yeast and bacteria from addition of NaCl, sugar, ethanol, and low pH

**Figure 5:**
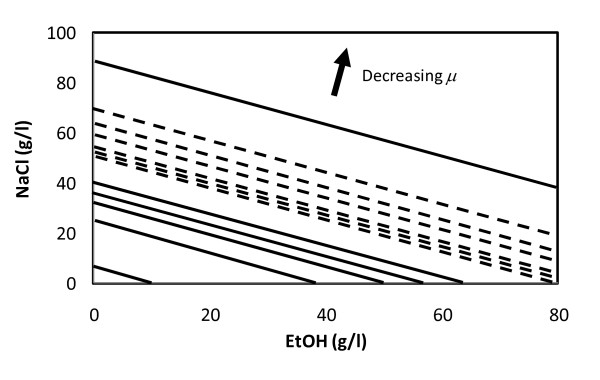
**Predicted limits of ethanol and NaCl concentrations at which the specific growth rate equals 0.1 h^-1 ^of yeasts (dashed lines) and lactic acid bacteria (solid lines)**. The area above each line indicate combinations of NaCl and ethanol concentrations leading to growth rates below 0.1 h^-1 ^for (top to bottom) *Lactobacillus plantarum *ATCC14431, commercially available baker's yeast (Jästbolaget AB, Sollentuna, Sweden), *Saccharomyces cerevisiae *CCUG53310, diploid *S. cerevisiae *X2180aα, diploid *S. cerevisiae *CEN.PK122, haploid *S. cerevisiae *X2180-1A, haploid *S. cerevisiae *CEN.PK113-7D, *Lactobacillus fermentum *ATCC14931, *Lactobacillus paracasei *ATCC25598, and industrial isolates of *Lactobacillus buchneri*, *Lactobacillus pantheris and L. plantarum *from Domsjö Fabriker, Örnsköldsvik, Sweden. Growth rates were estimated by multiple regression analysis of results from 435 batch cultures on rich media (yeast extract/peptone/dextrose (YPD) for yeast, MRS for bacteria). Growth rates were predicted at T = 30°C, pH = 5, lactic acid concentration 4 g/l, and glucose concentration 100 g/l.

Due to the potentially detrimental effect of bacterial contaminations on yeast fermentations it was decided to assess whether it was possible to choose process conditions that would selectively inhibit bacteria with minimum effects on the yeast. A systematic study on the effect of low pH and additions of NaCl, sugar, ethanol on the growth of yeast and bacteria isolated from industrial ethanol production plants was performed. The results regarding stress tolerance in various yeast strains can be found in [14]. Concerning bacteria, more than 15 isolates of *Lactobacilli *as well as 3 strains reported to be relevant in similar environments, together with several isolates of *Acetobacter *were included in this investigation (Albers and Larsson, unpublished results). Of these, a subset of eight representative *Lactobacilli*, six yeast, and two *Acetobacter *strains were chosen for a wider analysis at several treatment levels in rich yeast extract/peptone/dextrose (YPD) or MRS media. These screening experiments were performed using pure cultures. The results were evaluated using multiple regression analysis. No single parameter could be expected to selectively favor yeast at the expense of bacteria. However, the fitted response surfaces (Figure 5) indicated that *Lactobacilli *may be more sensitive than yeast to combinations of elevated NaCl and ethanol concentrations. The *Acetobacter *strains grew very slowly in this experimental set up in comparison to the *Lactobacilli *and yeasts in all tested conditions.

### Identification of optimum combinations of NaCl + ethanol selectively inhibiting bacteria

**Figure 6:**
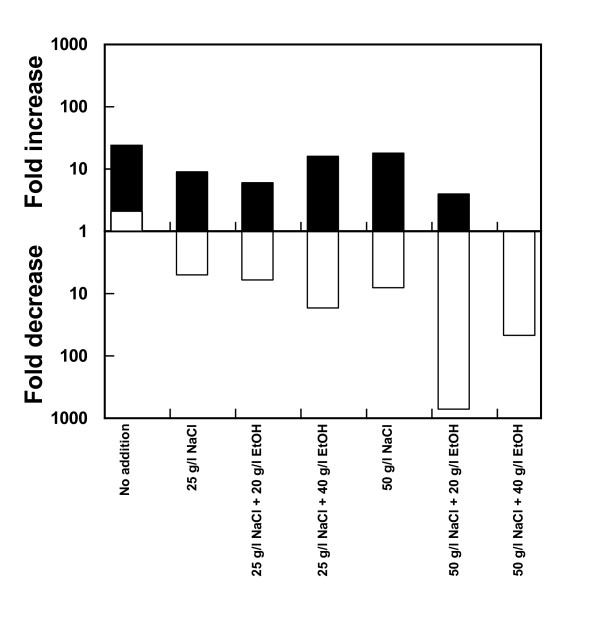
**Fold change in viable yeast (closed bars) and viable bacteria (open bars) after 24 h incubation in lignocellulosic medium with and without additions of ethanol and/or NaCl**. The fold change of viable yeast and bacteria was calculated after 24 h incubation in falcon tubes of a mixture of the yeast *Saccharomyces cerevisiae *and the bacteria *Lactobacillus buchneri, Lactobacillus fermentum, Acetobacter syzygii *and *Acetobacter tropicalis *inoculated into a dilute acid spruce hydrolysate medium with and without various additions of ethanol and/or NaCl. A missing bar means that the change is lower than onefold.

In order to test the hypothesis that enhanced levels of NaCl together with ethanol could selectively inhibit bacteria, a number of cocultures with yeast, *L. buchneri, L. plantarum Acetobacter syzygii *and *Acetobacter tropicalis *(all isolated from Domsjö Fabriker AB) were performed using a variety of combinations between NaCl and ethanol (Figure 6). To evaluate the effectiveness of this treatment a calculation was performed after 24 h of incubation concerning the increase in viable yeast cells and decrease in viable bacteria, respectively. The conditions finally chosen for more rigorous testing were 25 g/l NaCl + 40 g/l ethanol and 50 g/l NaCl + 20 g/l ethanol, since these combinations resulted in the largest decrease in bacterial viability while yeast could still maintain its viability. The former condition resulted in an increase in viable yeast cells almost similar to the increase obtained by the control without additions while the number of bacteria showed a more than tenfold decrease (Figure 6). Raising the NaCl concentration to 50 g/l and reducing ethanol to 20 g/l provoked a very drastic reduction in viable bacteria, close to 1,000-fold, while there was still an increase in viability of yeast during the 24 h period of testing (Figure 6).

### Enhancing yeast viability and productivity by a combination of NaCl and ethanol additions

**Figure 7:**
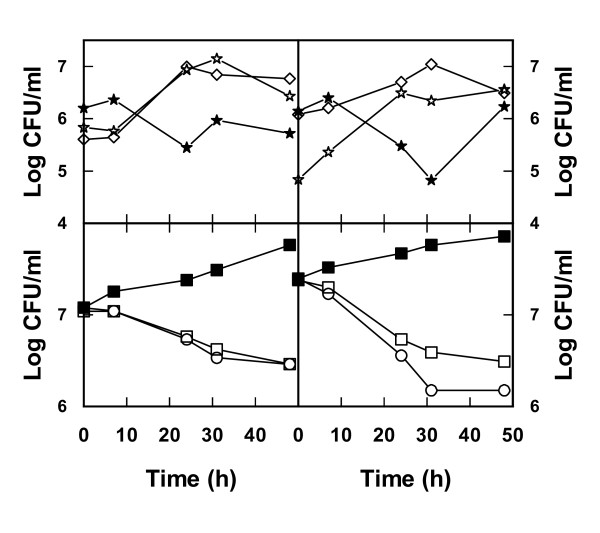
**Viability of yeast (upper panels) and bacteria (lower panels) during incubation in lignocellulosic medium with and without addition of ethanol and NaCl**. Lignocellulosic dilute acid spruce hydrolysate medium was inoculated with a mixture of the yeast *Saccharomyces cerevisiae *and the bacteria *Lactobacillus buchneri, Lactobacillus plantarum, Acetobacter syzygii *and *Acetobacter tropicalis*. Viability was measured without additions (filled stars, filled squares) and with addition of 40 g/l of ethanol and 25 g/l of NaCl (open stars, open squares) or 20 g/l of ethanol and 50 g/l of NaCl (open diamonds, open circles). Results from two independent bioreactor cultures are shown.

**Figure 8:**
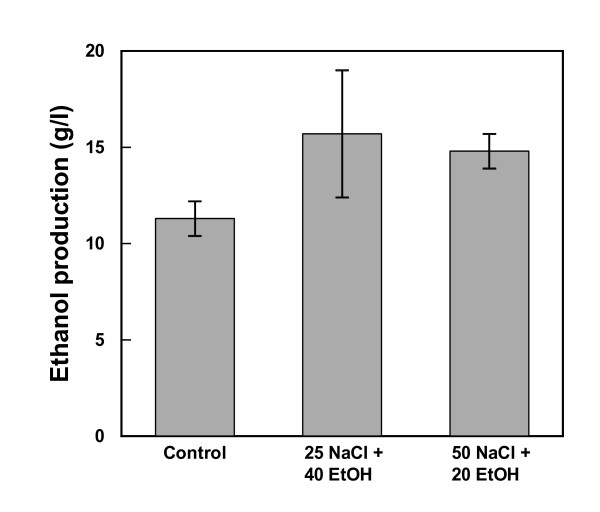
**Ethanol production in cocultures of yeast and bacteria incubated in lignocellulosic medium with and without addition of ethanol and NaCl**. Lignocellulosic dilute acid spruce hydrolysate medium without or with additions of 40 g/l of ethanol and 25 g/l of NaCl or 20 g/l of ethanol and 50 g/l of NaCl was inoculated with a mixture of the yeast *Saccharomyces cerevisiae *and the bacteria *Lactobacillus buchneri, Lactobacillus plantarum, Acetobacter syzygii *and *Acetobacter tropicalis*. The ethanol concentration was measured after glucose exhaustion. Values represent data from two separate bioreactor cultures each and error bars indicate minimum/maximum values.

The successful treatment in terms of increased yeast viability and reduction of bacteria by these additions were verified by cultivations of cocultures in bioreactors. In the absence of NaCl and ethanol the bacteria multiplied in the lignocellulosic medium while yeast viability showed a negative trend (Figure 7). A totally opposite effect was seen on addition of NaCl + ethanol, that is, there was a substantial decrease in bacteria and improved growth of yeast (Figure 7). Also in terms of ethanol production, NaCl + ethanol additions turned out to be successful as there was indeed an increase in the amount of ethanol produced compared with results obtained using lignocellulosic medium without these additions (Figure 8). To some extent these results were somewhat surprising since both NaCl as well as ethanol additions in pure cultures also showed a negative effect on the yeast *S. cerevisiae*. Apparently, reducing the viability of bacteria is crucial to having a net positive effect on the yeast performance in these mixed cultures.

### Verification of laboratory results in an industrial ethanol production plant

**Figure 9:**
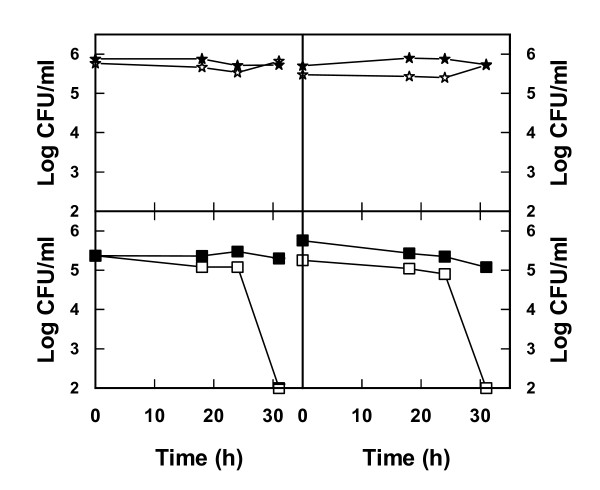
**Viability of yeast (upper panels) and bacteria (lower panels) in a sludge obtained from an industrial ethanol production unit with and without additions of ethanol and NaCl**. Sludge with its natural microbial flora of yeast and bacteria, obtained from an ethanol production plant (Domsjö Fabriker, Örnsköldsvik, Sweden), was used for inoculating spent sulfite liquor medium. Viability of yeast and bacteria was assessed for non-treated samples (closed symbols) and samples subjected to addition of 12.5 g/l of ethanol and 25 g/l of NaCl (open symbols). Results from two entirely different cultivations are shown.

In order to test whether additions of NaCl together with ethanol could be a strategy to reduce the amount of bacteria also at a real industrial ethanol production plant, experiments were carried out at Domsjö Fabriker AB in Örnsköldsvik, Sweden. Samples were obtained from the production plant at this site and various combinations of NaCl/ethanol additions were tested. It should be kept in mind that this kind of test will include the response of the entire microbial community at the selected site and not only some selected isolates. Preferably, these tests should be repeated at different time points as the conditions and composition of the microbial flora change over time. As it turned out it was indeed possible to restrict bacterial contaminants by additions of NaCl and ethanol in a real industrial environment (Figure 9). However, it did require extensive testing and fine tuning of the concentrations used, and in our case a combination of 25 g/l NaCl and 12.5 g/l ethanol added was the most effective combination (Figure 9).

## Conclusions

Acetic acid bacteria can potentially be a much more serious threat than lactic acid bacteria as contaminants of industrial scale yeast fermentations. Acetic acid bacteria showed a capacity to drastically reduce the viability of yeast cells as well as consuming the previously formed ethanol. Lactic acid bacteria did not show any of these characteristics. A combined addition of NaCl and ethanol during cultivation in wood hydrolysate could be used to reduce the number of bacteria and to selectively support the viability of yeast cells and thereby increase the concentration of ethanol.

A strategy to implement this in an industrial setting could be to add ethanol by recycling of process streams and to start the process using only a fraction of the total volume. This will potentially offer a kick-start for yeast in comparison to bacteria and preclude the necessity of adding large total amounts of NaCl and EtOH.

## Methods

### Strains

An industrial strain of *S. cerevisiae *was used (CCUG53310, Culture Collection University of Göteborg, Göteborg, Sweden) [15]. This strain was originally isolated from an industrial ethanol production plant, Domsjö Fabriker AB, Örnsköldsvik, Sweden. Lactic acid bacteria were obtained from a culture collection, *Lactobacillus fermentum *(ATCC14931) or isolated from Domsjö Fabriker AB, *L. buchneri*, *L. plantarum*. Acetic acid bacteria, *Acetobacter tropicalis*, *A. syzygii*, were isolated from the same industrial plant. The isolated bacteria were species determined by a combination of API test (bioMerieux, France) and 16S RNA gene sequencing.

### Cultivations

Inoculum cultures were grown in YPD medium (10 g/l yeast extract (Sigma-Aldrich St. Louis, USA), 20 g/l peptone (Nordic Biolabs, Taby, Sweden), 20 g/l glucose (Merck, Darmstadt, Germany)) for yeast and MRS medium (Oxoid, Hampshire, England) for bacteria for 1 to 1.5 days at 30°C in falcon tubes or shake flasks depending on culture volume.

The cultivations were performed in a lignocellulosic hydrolysate of spruce chips pretreated with dilute acid, a composition determined previously [14]. The pH was adjusted to 5.0 with ammonia (Merck, Darmstadt, Germany) and the hydrolysate medium was filter sterilized before usage.

For cocultivations on a small scale in 50 ml falcon tubes, the cells in the inocula were harvested after 1 day by centrifugation, resuspended in sterile water and the optical density at 610 nm (OD_610_) was measured. The hydrolysate (6 or 10 ml) was inoculated with cells (yeast and/or bacteria) and water in a total of 45 μl/ml hydrolysate to give an initial OD_610 _of 0.05 for yeast and at 0.09 for bacteria. The lid was closed and the tubes were incubated at 30°C in a rotary shaker and monitored for up to 4 days. Samples for medium analyses were centrifuged (2 min, at minimum 14,000 *g*) and stored at -20°C before analysis.

Cocultivations on a large bench scale were performed with 1 l of medium using 3 l bioreactors (Belach Bioteknik AB, Stockholm, Sweden) operated at 30°C with a stirring rate of 300 rpm and no gas inlet. The initial pH was set to 5.0 with ammonia (Merck, Darmstadt, Germany) during the preparation of medium and the decrease during cultivations was always less than 0.5 pH units.

### Determination of bacteria and yeast viability at an industrial production plant

The start inoculum was a mixture of microorganisms harvested from the Domsjö Fabriker industrial ethanol production plant located in Örnsköldsvik, Sweden. This mixture (sludge) contained the complete microbiological community existing in an industrial ethanol fermentation plant: mainly yeast, lactic acid bacteria and acetic acid bacteria.

This microbiological community was cultivated for 32 h at 30°C in spent sulfite liquor supplemented with 10.2 ml 25% ammonium (Merck, Darmstadt, Germany) and 171 mg/l KH_2_PO_4 _(Merck, Darmstadt, Germany) with and without addition of NaCl (Merck, Darmstadt, Germany) (25 g/l) + ethanol (VWR, Leuven, Belgium) (12.5 g/l). The pH was adjusted to 5.0 by addition of 5 M NaOH (Merck, Darmstadt, Germany) prior to fermentation. The cultivations were performed in 300 ml Erlenmeyer flasks with a total volume of 200 ml.

Measurements of the cell viability were performed by colony forming unit (CFU) count.

### Analyses

Metabolites in the medium (ethanol, acetic acid, lactic acid) were analyzed using commercial enzymatic kits assays (R-Biopharm GmbH, Darmstadt, Germany) with adapted volumes in microtiter plates. Absorbance was measured with a Fluostar Galaxy plate reader (BMG Labtechnologies, Offenburg, Germany).

CFU determinations were performed on agar plates with YPD for yeast (when bacteria was present in large numbers, 20 μl of 50 g/l ampicillin (AppliChem, Darmstadt, Germany) was added to each plate) and MRS for bacteria with 0.1 g/l cycloheximide (Merck, Darmstadt, Germany)to suppress growth of yeast. Each dilution was spread on two or three plates. The plates were incubated at 30°C for 2 days for yeast and for 3 days for bacteria to establish distinct colonies before counting.

Multiple regression analysis of the specific growth rate as a function of pH, temperature, and concentrations of NaCl, glucose, ethanol and lactic acid was performed using the software Modde 9.0 (Umetrics AB, Umea, Sweden).

## Competing interests

The authors declare that they have no competing interests.

## Authors' contributions

EA participated in the design of the study, performed all the laboratory scale experiments and took part in interpretation of results and writing of the manuscript. EJ participated in the design of and performed all the experiments at industrial scale, and took part in interpretation of results and writing of the manuscript. CJF performed the multiple regression analysis and took part in interpretation of results and writing of the manuscript. CL designed the study, took part in interpretation of results and was the main author. All authors read and approved the final manuscript.
